# Diversity of SCC*mec* Elements in Methicillin-Resistant Coagulase-Negative Staphylococci Clinical Isolates

**DOI:** 10.1371/journal.pone.0020191

**Published:** 2011-05-26

**Authors:** Zhiyong Zong, Chunhong Peng, Xiaoju Lü

**Affiliations:** 1 Center of Infectious Diseases, West China Hospital, Sichuan University, Chengdu, China; 2 Department of Respiratory Medicine, West China Hospital, Sichuan University, Chengdu, China; 3 Division of Infectious Diseases, State Key Laboratory of Biotherapy, Chengdu, China; 4 Department of Emergence, People's Hospital of Guizhou Province, Guiyang, China; East Carolina University, United States of America

## Abstract

**Background:**

Methicillin-resistant coagulase-negative staphylococci (MR-CoNS) are opportunistic pathogens and serve as a large reservoir of staphylococcal cassette chromosome *mec* (SCC*mec*). Characterization of SCC*mec* in MR-CoNS can generate useful information on the mobilization and evolution of this element.

**Methodology/Principal Findings:**

Non-repetitive MR-CoNS clinical isolates (n = 84; 39 *S. epidermidis*, 19 *S. haemolyticus*, 9 *S. hominis*, 6 *S. capitis*, 4 *S. warneri*, 2 *S. cohnii*, 2 *S. saprophyticus*, 1 *S. kloosii*, 1 *S. simulans* and 1 *S. massiliensis*) were collected. All isolates could grow on plates with 4 mg/L cefoxitin and all had *mecA* as detected by PCR. Strain typing using RAPD and ERIC-PCR revealed that almost all isolates were of different strains. SCC*mec* typing was performed using multiplex PCR published previously. For isolates in which SCC*mec* could not be typed, the *mec* complex classes were determined by additional PCR and the *ccr* genes were amplified with published or newly-designed primers and then sequenced. SCC*mec* types were assigned for 63 isolates by multiplex PCR and were assigned for 14 other isolates by PCR targeting *mec* and *ccr*. Among 77 isolates with determined SCC*mec* types, 54 had a single type, including type III (n = 19), IV (n = 14), V (n = 10), II (n = 2), I (n = 1), VIII (n = 1) and five unnamed types (n = 7), while 23 isolates had two types, III+V (n = 12), II+V (n = 8), II+IV (n = 2) or IV+V (n = 1). The five unnamed types were assigned UT1 (class A *mec*, *ccrA1*/*ccrB4*), UT2 (class C1 *mec*, *ccrA4/ccrB4*), UT3 (class A *mec*, *ccrA5/ccrB3*), UT4 (class C2 *mec*, *ccrA2/ccrB2* plus *ccrC1*) and UT5 (class A *mec*, *ccrA1/ccrB1* plus *ccrC1*).

**Conclusions/Significance:**

SCC*mec* types III, IV and V were prevalent in MR-CoNS and many isolates could harbor more than one type. Several new types of SCC*mec* were identified, highlighting the great genetic diversity and the need of developing classification schemes for SCC*mec* in MR-CoNS.

## Introduction

Coagulase-negative staphylococci (CoNS) comprise a variety of *Staphylococcus* species and are opportunistic pathogens commonly associated with infections in patients with indwelling devices or being immunocompromised [1]. CoNS are usually resistant to methicillin [2] and in staphylococci, methicillin resistance is mainly due to the expression of the *mecA* gene, which specifies penicillin binding protein 2a (PBP2a), a transpeptidase with a low affinity for β-lactams [3], [4]. *mecA* is carried by a mobile genetic element (MGE) termed the staphylococcal cassette chromosome *mec* (SCC*mec*) [5]. Generally, SCC*mec* contains two essential components, i.e. the *mec* gene complex and the *ccr* gene complex. The *mec* gene complex consists of *mecA*, the regulatory genes and associated insertion sequences and has been classified into six different classes, i.e. A, B, C1, C2, D and E (Table 1). Cassette chromosome recombinase (*ccr*) genes (*ccrC* or the pair of *ccrA* and *ccrB*) encode recombinases mediating integration and excision of SCC*mec* into and from the chromosome [6], [7]. The *ccr* gene(s) and surrounding genes form the *ccr* gene complex. In addition to *ccr* and *mec* gene complexes, SCC*mec* contains a few other genes and various other MGE, e.g. insertion sequences, transposons and plasmids [7].

**Table 1:** SCC*mec* types.

SCC*mec*type^1^	*mec* class^2^	*ccr*type^3^	Species (locations and references)
**I**	B	A1B1	
**II**	A	A2B2	
**III**	A	A3B3	
**IV**	B	A2B2	
**V**	C2	C1	
VI	B	A4B4	
VII	C1	C1	
**VIII**	A	A4B4	
IX	C2	A1B1	
X	C1	A1B6	
XI	E	A1B3	
**UT1**	A	A1B4	*S. saprophyticus* (China, this study)
**UT2**	C1	A4B4	*S. haemolyticus* (China, this study), *S. simulans* (China, this study)
**UT3**	A	A5B3	*S. hominis* (China, this study), *S. cohnii* (China [34]), *S. pseudintermedius* (Switzerland [19])
**UT4**	C2	A2B2&C1	*S. epidermidis* (China, this study; Algeria, Cambodia and Mali [8])
**UT5**	A	A1B1&C1	*S. cohnii* (Algeria [8]), *S. hominis* (China, this study)
UT5v	A	A1B1	*S. capitis* (Finland [27]), *S. hominis* (Finland [27], Norway [28])
UT6	A	C1	*S. epidermidis* (Finland [27]; Mali [8])
UT7	B	C1	*S. epidermidis* (Algeria [8]), *S. hominis* (Mali [8])
UT8	C2	A4B4&C1	*S. epidermidis* (Algeria [8])
UT9	C1	A5B3^4^	*S. haemolyticus* (China [11])
UT10	C1	A2B2&C1	*S. haemolyticus* (Norway [28])

1 SCC*mec* types I to XI have been assigned for *S. aureus* according to http://www.sccmec.org/Pages/SCC_TypesEN.html. UT1 to UT10 types are assigned in this study. The types seen in this study are bolded. UT5v represents a variant of UT5.

2 Class of *mec*: A, IS*431*-*mecA*-*mecR1*-*mecI*; B, IS*431*-*mecA*-*ΔmecR1*-IS*1272*; C1, IS*431*-*mecA*-*ΔmecR1*-IS*431* (two IS*431* in the same direction); C2, IS*431*-*mecA*-*ΔmecR1*-IS*431* (two IS*431*s in the opposite direction); D, IS*431*-*mecA*-*ΔmecR1*; E, *blaZ*-*mecA*_LGA251_-*mecR1*_LGA251_-*mecI*_LGA251_.

3 *ccr* type: A, *ccrA*; B, *ccrB*; C1, *ccrC1*.

4 originally designated *ccrA*_SHP_ and *ccrB*_SHP_.

Eleven types (I to XI) of SCC*mec* have been assigned for *Staphylococcus aureus* based on the classes of the *mec* gene complex and the *ccr* gene types (www.sccmec.org/Pages/SCC_TypesEN.html) (Table 1). As methicillin resistance is prevalent in CoNS, methicillin-resistant CoNS (MR-CoNS) may serve as a large reservoir of SCC*mec* available for *S. aureus* to form methicillin-resistant *S. aureus* (MRSA) [6]. According to the available data [8], [9], [10], [11], [12], [13], [14], [15], [16], [17], [18], [19], SCC*mec* elements are more diverse in MR-CoNS, with new variants of *ccr* genes continuing to be identified [11], [18], [19], [20]. In addition, many SCC*mec* elements in MR-CoNS could not be typed using currently-available schemes based on multiplex PCR [6], [19]. To obtain the information on SCC*mec* in local MR-CoNS, 84 clinical isolates were investigated.

## Methods

### Bacterial isolates

Non-repetitive MR-CoNS isolates were collected from clinical specimens in West China Hospital, Chengdu, western China, from March to May 2010. Species identification and antimicrobial susceptibility were determined using the MicroScan Walkaway 96 SI (Siemens Healthcare Diagnostic, Deerfield, IL) or Vitek II (bioMérieux, Durham, NC) automated microbiology system. These isolates could also grow on brain heart infusion plates (Oxoid, Hampshire, UK) containing 4 mg/L cefoxitin (Sigma, St Louis, MO). Species identification of 20 randomly-selected isolates was confirmed by partially sequencing 16S rRNA genes amplified with the universal primers 27F and 1492R [21].

### Strain typing

MR-CoNS were typed using two rapid PCR-based methods, i.e., random amplification of polymorphic DNA (RAPD) with the 10-mer primer (5′-AGCGTCACTG-3′) and the Enterobacterial Repetitive Intergenic Consensus (ERIC) PCR with the primer pair of ERIC 1R and ERIC 2 as descried previously [22], [23]. Isolates were considered of the same strain if they had identical RAPD patterns and identical ERIC-PCR profiles; otherwise, they would be assigned to different strains.

### SCC*mec* typing

Genomic DNA of MR-CoNS isolates were prepared using a commercial kit (Tiangen, Beijing, China) and then used as template for PCR. Maxima Hot Start Taq (Fermentas, Burlington, ON, Canada) and ExTaq premix (Takara, Dalian, China) were used for multiplex and singlex PCR, respectively. SCC*mec* typing was performed using the multiplex PCR scheme that was published previously [24]. For isolates in which SCC*mec* could not be typed by the multiplex PCR, classes of the *mec* complex and the *ccr* genes (*ccrAB1*, *ccrAB2*, *ccrAB3* and *ccrC1*) were examined by additional PCR as described previously [24]. For those that primers targeting *ccrAB1*, *ccrAB2*, *ccrAB3* and *ccrC1* [24] failed to yield amplicons, additional published or newly-designed primers (listed in Table 2) were used to amplify *ccrA*, *ccrB*, *ccrC1* and *ccrC2* genes and amplicons were then sequenced. *ccr* allotypes were assigned based on >85% nucleotide identity with known allotypes [7]. For those that did not yield amplicons from PCR amplifying class A or B *mec* complex, the presence of the insertion sequence IS*431* upstream and downstream of *mecA* was examined by PCR using a primer (IS431-F2 or IS431-R1, Table 2) located in either direction of IS*431* paired with a primer in *mecA*. SCC*mec* types were assigned based on the *mec* complex classes and the *ccr* gene types according to the criteria set for *S. aureus* [7].

**Table 2:** Selected primers used for PCR.

Primer	Sequence (5′-3′)^1^	Target/location	Reference
ccrA-UF1	AATGTGAHGTATTATGTTGYTA	*ccrA*	[34]
ccrA-UR1	GGTTCATTTTTDAARTAGAT		
ccrA-UF2	AYTHCATCGYAAYYTGAAAAA	*ccrA*	This study
ccrA-UR2	ACGDCCACARTAGTTAGGRTT		This study
ccrA_up	TGCATTCATGTTTTGAGGAC	*ccrA*	[11]
ccrA_dw	CAATGTGACGTATTGTGTTG		
ccrB-UF1	CGTGTATCAACDGAAATVCAA	*ccrB*	[34]
ccrB-UR1	CTTTATCACTTTTGAYWATTTC		
ccrB_up	GTTCCTTTACCATGGACTTG	*ccrB*	[11]
ccrB_dw	CTAGAAGGCTACTATCAAGG		
ccrC-UF1	GCAATGAAACGTCTATTACAA	*ccrC1*	This study
ccrC-UR1	TTTCATCRATAACYAAATCA		
28-24	GGAACAATCAGAGCGTGGA	*ccrC2*	[34]
28-26	ACGTTTCACAGCCCAATTTT		
IS431-F2	GGTCTACCGTTGGGTTCAAG	IS*431*	This study
IS431-R1	CGTCTCATCAATACGCCATTT	IS*431*	This study
mecA-R2	TCGGACGTTCAGTCATTTCT	*mecA*	This study

^*1*^D: A, G or T; H: A, C or T: M: A or C; R: A or G; W: A or T; Y: C or T; V: A, C or G.

### Sequencing

Amplicons were purified using a commercial kit (Omega, Norcross, GA) and then sequenced using an ABI 3730xl DNA Analyzer (Applied Biosystems, Foster City, CA) at the Beijing Genomics Institute (Beijing, China). Sequences were assembled using the SeqMan II program in the Lasergene package (DNASTAR Inc, Madison, WI) and similarity searches were carried out using BLAST programs (http://www.ncbi.nlm.nih.gov/BLAST/).

#### Nucleotide accession numbers

The sequences of new *ccr* variants have been deposited at GenBank under accession numbers HQ848693 to HQ848707.

## Results

### MR-CoNS isolates were diverse in clonality

A total of 84 MR-CoNS were collected from clinical specimens, including 39 *Staphylococcus epidermidis*, 19 *Staphylococcus haemolyticus*, 9 *Staphylococcus hominis*, 6 *Staphylococcus capitis*, 4 *Staphylococcus warneri*, 2 *Staphylococcus cohnii*, 2 *Staphylococcus saprophyticus*, 1 *Staphylococcus kloosii*, 1 *Staphylococcus simulans* and 1 *Staphylococcus massiliensis* (Table 3). Of note, *S. massiliensis* is a newly-recognized species [25]. To our knowledge, as *S. massiliensis* has only been reported once before, this study is therefore probably the second ever report of this species.

**Table 3:** SCC*mec* typing results.

Species	No.	Origins (no.)^1^	SCC*mec* type^2^ (no.)
*S. epidermidis*	39	Wound secretion (25), blood (8), ascites (2), CSF (2), drainage (1), urine (1)	I (1), II (1), III (8), IV (11), V (5), II+V (1), III+IV (1), III+V (8), IV^3^+V (1), UT4 (1), NT (1)
*S. haemolyticus*	19	Wound secretion (7), blood (2), prostatic fluid (4), ear secretion (1), CSF (1), urine (2), urethral meatus secretion (1), sputum (1)	II (1), III (4), V (3), II+V (6), III+V (1), UT2 (2), NT (2)
*S. hominis*	9	Blood (6), wound secretion (2), CSF (1)	III (3), V (1), VIII (1), II+V (1), UT3 (1), UT5 (1), NT (1)
*S. capitis*	6	Wound secretion (2), blood (2), prostatic fluid (1), urine (1).	III (1), IV (1), III+IV (1), III+V (3)
*S. warneri*	4	CSF (3), wound secretion (1)	IV (2), III (1), NT (1)
*S. cohnii*	2	Blood (1), wound secretion (1)	III (2)
*S. saprophyticus*	2	Blood (2)	UT1 (1), NT (1)
*S. kloosii*	1	Wound secretion (1)	NT (1)
*S. simulans*	1	Sputum (1)	UT2 (1)
*S. massiliensis*	1	Wound secretion (1)	V (1)
Total	84	Wound secretion (40), blood (21), CSF (7), urine (4), prostatic fluid (5), ear secretion (1), ascites (2), sputum (2), drainage (1), urethral meatus secretion (1)	I (1), II (2), III (19), IV (14), V (10), VIII (1), II+V (8), III+IV (2), III+V (12), IV+V (1), UT1 (1), UT2 (3), UT3 (1), UT4 (1), UT5 (1), NT (7)

1 CSF, cerebral spinal fluid;

2 SCC*mec* types (the *ccr* type and class of the *mec* complex) is available in Table 1.

3 The isolate was positive to PCR specific for two IV subtypes, IVa and IVb, in addition to V.

These 84 isolates had a quite diverse clonal background. For species other than *S. epidermidis* and *S. haemolyticus*, none of isolates had identical RAPD and ERIC profiles (data not shown) and these isolates were therefore of different strains. *S. epidermidis* isolates (n = 39) could be assigned to 34 strains with five isolates from two wards (3 from orthopedics and 2 from neurology) belonging to a single strain in light of their identical RAPD and ERIC profiles. A total of 17 strains could be identified for the 19 *S. haemolyticus* isolates. Two *S. haemolyticus* isolates from different wards (neurosurgery and endocrinology) belonged to a single strain and two other isolates (from orthopedics and neurology) were also of a single strain. Although nosocomial dissemination of individual *S. epidermidis* and *S. haemolyticus* strains was identified, the vast majority of MR-CoNS isolates from different patients were of different strains and no strain could be identified as the predominant clone circulating locally. This suggests that most isolates might not have been acquired in the hospital.

### SCC*mec* types were determined by multiplex PCR in most MR-CoNS isolates

All of the 84 MR-CoNS isolates had *mecA* as detected by PCR [24]. SCC*mec* types were assigned for 63 of 84 (75%) isolates by multiplex PCR. Among these 63 isolates, 40 had a single SCC*mec* type including type I (n = 1), II (n = 1), III (n = 15), IV (n = 13; IVa subtype, n = 12, and IVd subtype, n = 1) and V (n = 10), while 23 had two types including II+V (n = 8), III+IV (n = 2), III+V (n = 12) and IV+V (n = 1, containing two IV subtypes, IVa and IVb).

### Five unnamed types were identified

The remaining 21 isolates in which SCC*mec* types could not be determined by multiplex PCR, additional PCR amplifying the *mec* complexes and *ccr* genes and sequencing were used to determine SCC*mec* types. SCC*mec* types could be assigned for 14 of the 21 MR-CoNS (Table 4), including Type II for 1 isolate (*S. epidermidis*), Type III for 4 (2 *S. haemolyticus*, 1 *S. hominis* and 1 *S. cohnii*), Type IV for 1 (*S. epidermidis*), Type VIII for 1 (*S. hominis*) and five unnamed types for 7. Of note, Type VIII SCC*mec* was originally found in MRSA [26] and has rarely been reported in MR-CoNS except that a variant of Type VIII (class A *mec*, *ccrA4*/*ccrB4* plus *ccrC1*) have been found in three *S. epidermidis* from Finland [27].

**Table 4:** Closest matches of *ccrA* and *ccrB* genes in the 14 isolates in which SCC*mec* types were determined by PCR amplifying the *mec* complexes and *ccr* genes.

Isolate	Species	SCC*mec*type	*ccr*variant	Closest match in *S. aureus* *ccr* (identity %); strain	Closest match in CoNS*ccr* (identity %); species strain
WCG53	*S. epidermidis*	II	*ccrA2*	*ccrA2* (95.5); M06/0075	*ccrA2* (95.5); *S. epidermidis* RP62A
			*ccrB2*	*ccrB2* (97.0); JCSC1968	*ccrB2* (97.5); *S. epidermidis* CS8
WCF34	*S. haemolyticus*	III	*ccrA3*	*ccrA3* (100); JKD6008	*ccrA3* (100); *S. pseudintermedius* KM1381
			*ccrB3*	*ccrB3* (100); JKD6008	*ccrB3* (100); *S. pseudintermedius* KM1381
WCG74	*S. hominis*	III	*ccrA3*	*ccrA3* (100); JKD6008	*ccrA3* (100); *S. pseudintermedius* KM1381
			*ccrB3*	*ccrB3* (99.9); JKD6008	*ccrB3* (99.9); *S. pseudintermedius* KM1381
WCH47	*S. hominis*	III	*ccrA3*	*ccrA3* (98.2); JKD6008	*ccrA3* (98.3); *S. cohnii* WC28
			*ccrB3*	*ccrB3* (100); TW20	*ccrB3* (99.9); *S. pseudintermedius* KM1381
WCH62	*S. cohnii*	III	*ccrA3*	*ccrA3* (94.7); JKD6008	*ccrA3* (100); *S. cohnii* WC28
			*ccrB3*	*ccrA3* (82.5); JKD6008	*ccrB3* (99.9); *S. cohnii* WC28
WCH08	*S. epidermidis*	IV	*ccrA2*	*ccrA2* (99.7); JCSC6668	*ccrA2* (99.2); *S. epidermidis* RP62A
			*ccrB2*	*ccrB2* (100); JCSC1968	*ccrB2* (98.3); *S. epidermidis* ATCC 12228
WCG25	*S. hominis*	VIII	*ccrA4*	*ccrA4-2* (100); CHE482	*ccrA4* (81.7); *S. epidermidis* ATCC 12228
			*ccrB4*	*ccrA4-2* (100); CHE482	*ccrB4* (91.7); *S. epidermidis* ATCC 12228
WCH02	*S. saprophyticus*	UT1	*ccrA1*	*ccrA1* (99.5); COL	*ccrA1* (99.7); *S. hominis* GIFU12263
			*ccrB4*	*ccrA4-2* (100); CHE482	*ccrB4* (91.7); *S. epidermidis* ATCC 12228
WCF77	*S. haemolyticus*	UT2	*ccrA4*	*ccrA4-1* (94.1); CHE482	*ccrA4* (90.5); *S. epidermidis* ATCC 12228
			*ccrB4*	*ccrB4-1* (94.4); CHE482	*ccrB4* (94.0) ;*S. epidermidis* ATCC 12228
WCG38	*S. haemolyticus*	UT2	*ccrA4*	*ccrA4-1* (94.0); CHE482	*ccrA4* (90.4); *S. epidermidis* ATCC 12228
			*ccrB4*	*ccrB4* (93.8); BK20781	*ccrB4* (93.8); *S. epidermidis* ATCC 12228
WCH55	*S. simulans*	UT2	*ccrA4*	*ccrA4-1* (95.8); CHE482	*ccrA4* (93.0); *S. epidermidis* ATCC 12228
			*ccrB4*	*ccrB4* (94.3); HDE288	*ccrB4* (93.5); *S. epidermidis* ATCC 12228
WCG08	*S. haemolyticus*	UT3	*ccrA5*	*ccrA1* (79.2); COL	*ccrA5* (85.3); *S. pseudintermedius* KM241
			*ccrB3*	*ccrB3* (83.2); RN7170	*ccrB3* (96.8); *S. cohnii* WC28
WCF80	*S. epidermidis*	UT4	*ccrA2*	*ccrA2* (98.6); N315	*ccrB2* (98.1); *S. epidermidis* RP62A
			*ccrB2*	*ccrB2* (98.6); JCSC1968	*ccrB2* (99.5); *S. epidermidis* ATCC 12228
WCI20	*S. hominis*	UT5	*ccrA1*	*ccrA1* (99.5); 45394F	*ccrA1* (95.4); *S. hominis* GIFU12263
			*ccrB1*	*ccrB1* (98.0); COL	*ccrB1* (90.9); *S. saprophyticus* ATCC 15305

The unnamed types identified (Table 1) were assigned UT1, UT2, UT3, UT4 and UT5 with UT representing unnamed Type, respectively. Among them, to our knowledge, UT1 and UT2 have not been described elsewhere and were therefore novel types. UT3, UT4 and UT5 have been described in MR-CoNS before in several reports (Table 1) and a variant of UT5 (class A *mec*, *ccrA1/ccrB1* but without *ccrC1*) have also been identified in Finland [27] (Table 1). In addition to UT1 to UT5 seen in this study, several unnamed types of SCC*mec* have been reported by other investigators [8], [11], [27], [28], designated UT6, UT7, UT8, UT9 and UT10 here (Table 1).

*ccr* genes could not be obtained from the remaining 7 isolates despite repeated attempts and SCC*mec* types in these isolates were therefore undetermined. Among the 7 isolates, 4 isolates (3 *S. haemolyticus* and 1 *S. kloosii*) had class C1 *mec*, 1 (*S. epidermidis*) had class C2 *mec* and 1 (*S. hominis*) had class A *mec* but the class of *mec* could not be determined for the remaining 1 (*S. warneri*).

### A diversity of *ccr* variants were present

As mentioned above, among the 21 isolates in which SCC*mec* types could not be determined by multiplex PCR, *ccr* genes were obtained from 14 isolates and then sequenced (Table 4). Variants of 10 *ccr* allotypes, *ccrA1*, *ccrA2*, *ccrA3*, *ccrA4*, *ccrA5*, *ccrB1*, *ccrB2*, *ccrB3*, *ccrB4* and *ccrC1*, were identified. The combinations of these *ccr* allotypes laid a foundation of nine different SCC*mec* types seen here (Table 4). In particular, the combination of *ccrA1*/*ccrB4* had not been seen before.

## Discussion

### Genetic diversity of SCC*mec* in MR-CoNS imposes a great challenge for SCC*mec* typing

In total, SCC*mec* types were determined for most isolates (77/84) using multiplex PCR and singlex PCR targeting *ccr* and the *mec* gene complex. SCC*mec* in MR-CoNS exhibited substantial genetic diversity with new types continuously being identified. Consistent with previous reports [8], [11], [14], [27], [29], [30], type I and VIII SCC*mec* were rare and type VI, VII, IX, X and XI have not been identified in MR-CoNS yet, while type II, III, IV and V were relatively common. In this study, type III was the most common type being present in 33 of 84 MR-CoNS isolates either alone or combined with other types, followed by type V (31/84) and then by IV (17/84).

The distribution of different types of SCC*mec* in MR-CoNS varied depending on the host species and possibly on the geographical locations. It has been proposed that type IV has been preferentially associated with *S. epidermidis* [8], [29], [31] and type V dominates in *S. haemolyticus* [8]. Indeed, most (13 of 17) type IV SCC*mec* were seen in *S. epidermidis* here. Type III SCC*mec* was in particular widely distributed and has been found in *S. aureus* and a variety of CoNS species, although the mechanism responsible for this wide distribution has not been well understood. In this study, type III was the dominant type of SCC*mec* in *S. epidermidis* (17/39), *S. capitis* (5/6) and *S. cohnii* (2/2), while in *S. haemolyticus* (n = 19) the most common type was the combination of type II and V (6/19).

A substantial proportion of MR-CoNS isolates (n = 23) were determined to have two SCC*mec* elements by multiplex PCR. This is no surprise as the co-existence of two SCC*mec* elements appears to be common in MR-CoNS [29]. It is likely that the two SCC*mec* elements actually constitute a composite rather than two independent units. One of the limitations of the multiplex and singlex PCR-based schemes is the inability to differ composites from separate elements. Consistent with previous reports [15], [28], [29], [32], SCC*mec* types in a few MR-CoNS isolates could not be assigned by currently-available PCR-based methods. In most cases of “non-typeable” SCC*mec*, *ccr* genes could not be amplified although the class of the *mec* gene complex had been determined. *ccr* genes might be unrecognized types, be deleted or contain mutations in the primer-targeting regions in these “non-typeable” SCC*mec* [6]. The frequent identification of co-existed SCC*mec* and the presence of “non-typeable” elements represent great challenges for SCC*mec* typing in MR-CoNS.

### A classification scheme for SCC*mec* typing in MR-CoNS is required

The presence of a few unnamed types identified previously and in this study suggests that more studies on MR-CoNS are required to reveal the reservoir of SCC*mec* and also highlights the need of establishing classification schemes for SCC*mec* in MR-CoNS. An obvious option is to extend the current MRSA-focused SCC*mec* classification scheme to those in MR-CoNS and therefore transforms it into a scheme for SCC*mec* not based on the species of host isolates. Alternatively, a new classification scheme specific for SCC*mec* in MR-CoNS should be developed based on the MRSA-focused scheme.

### Dynamics of SCC*mec* within CoNS and possible horizontal transfer of SCC*mec* between CoNS and *S. aureus*

None of *S. haemolyticus* isolates belonging to the same strain carried the same type of SCC*mec* elements, while the five *S. epidermidis* isolates of the same strain carried type III (n = 1), V (n = 2), III+IV (n = 1) or III+V (n = 1) SCC*mec*. Isolates carrying different SCC*mec* elements were of the same strain and isolates carrying the same type of SCC*mec* elements belonged to different strains. The discrepancy between strain background and the SCC*mec* type carried might suggest frequent gain and loss of SCC*mec* elements by CoNS.

Generally, *ccrA* and *ccrB* genes identified in local MR-CoNS were not always closest to the counterparts in MR-CoNS isolated elsewhere but could be closer to those in MRSA (Table 4). This may illustrate that SCC*mec* in MR-CoNS remains under-characterized but also suggests possible horizontal transfers of SCC*mec* between MR-CoNS and MRSA.

Interestingly, *ccrA4* and *ccrB4* genes in *S. hominis* strain WCG25 were identical to the *ccrA4-2* and *ccrB4-2* in MRSA strain CHE482, which circulated among intravenous drug users in Zurich, Switzerland [33]. *ccrA4-2* had 86.4% and 83.2% nucleotide identity to *ccrA4* genes in MRSA strains HDE288 and C10682, respectively; while *ccrB4-2* had 93.2% and 91.8% identity to *ccrB4* in HDE288 and C10682, respectively. HDE288 and C10682 were the representative strains of Type VI and Type VIII SCC*mec*, respectively. In light of the relatively low identity with *ccrA*/*B4* genes in other MRSA strains and their absence in MRSA outside Switzerland, *ccrA/B4-2* might have an origin outside *S. aureus*. The presence of *ccrA/B4-2* in *S. hominis* found here suggests a possible origin of the two genes. Of note, *S. saprophyticus* strain WCH02 also had *ccrB4-2* but had *ccrA1* instead of *ccrA4-2*. This suggests that homologous recombination between different types of SCC*mec* might have occurred and then resulted in a novel combination of *ccrA* and *ccrB* genes.

Many of *ccrA*/*B* genes identified here were new variants with nucleotide differences from all known ones available in GenBank, although all of which could be assigned to known allotypes based on the cut-off value, 85% identity. Among the variants identified, a *ccrA5* gene with relatively low identity (≤85.3%) to known *ccrA5* variants was present in *S. hominis* strain WCG08. *ccrA5* was mainly seen in CoNS but was also recently found in *S. aureus* strain Msida_536245_Z661 (GU066221). The *ccrA5* genes in WCG08, *S. pseudintermedius* strain KM241 (AM904731), *S. haemolyticus* H9 (EU934095) and *S. cohnii* WC28 (GU370073) displayed less than 90% identity between each other, suggesting that the diversity of *ccrA5* genes in CoNS might be species-specific.

In summary, for most MR-CoNS (63/84; 75%) SCC*mec* types were assigned by the multiplex PCR [24], which was originally developed for SCC*mec* in MRSA, suggesting that this scheme could be a suitable starter method for SCC*mec* typing for MR-CoNS. For two thirds (n = 14) of the remaining 21 isolates, SCC*mec* types were assigned by PCR targeting the *mec* and *ccr* complexes. According to our experience, multiple pairs of primers for *ccr* genes should be used to maximize the possibility of obtaining *ccr* amplicons. The common types of SCC*mec* in MR-CoNS were II, III, IV and V, either alone or in various combinations and Type III was in particular common and widely distributed in a variety of species. Only SCC*mec* types seen in MRSA have been assigned a number (I to XI), which left many SCC*mec* types in MR-CoNS unnamed. Five of such unnamed types were encountered locally and were tentatively assigned UT1 (class A *mec*, *ccrA1*/*ccrB4*), UT2 (class C1 *mec*, *ccrA4/ccrB4*), UT3 (class A *mec*, *ccrA5/ccrB3*), UT4 (class C2 *mec*, *ccrA2/ccrB2* plus *ccrC1*) and UT5 (class A *mec*, *ccrA1/ccrB1* plus *ccrC1*). UT1 and UT2 were new types, while UT3, UT4 and UT5 have been seen in MR-CoNS before. The growing number of unnamed types reported highlights the need of developing classification system for SCC*mec* types in MR-CoNS, either incorporating them in the current system for those in MRSA or establishing a new system.
